# Antimicrobial Susceptibility Test for the Determination of Resistant and Susceptible *S. aureus* and *Enterococcus* spp. Using a Multi-Channel Surface Plasmon Resonance Device

**DOI:** 10.3390/diagnostics9040191

**Published:** 2019-11-15

**Authors:** Gulsum Ucak Ozkaya, Muhammed Zeki Durak, Isin Akyar, Onur Karatuna

**Affiliations:** 1 Department of Food Engineering, Faculty of Chemical and Metallurgical Engineering, Yildiz Technical University, Istanbul 34210, Turkey; 2 Department of Food Engineering, Faculty of Engineering and Architecture, Bitlis Eren University, Bitlis 13000, Turkey; 3 Department of Medical Microbiology, School of Medicine, Acibadem University, Istanbul 34752, Turkey; isinakyar@gmail.com (I.A.); onurkaratuna@gmail.com (O.K.)

**Keywords:** antimicrobial susceptibility, minimum inhibitory concentration assay, methicillin-resistant *S. aureus*, nosocomial infection, surface plasmon resonance, vancomycin-resistant *Enterococcus*

## Abstract

The objective of this study was to investigate the development of a surface plasmon resonance (SPR) sensor platform equipped with multiple channels for the simultaneous determination of methicillin-resistant *S. aureus* (MRSA), methicillin-susceptible *S. aureus* (MSSA) and vancomycin-resistant *Enterococcus* (VRE), and vancomycin-susceptible *Enterococcus* (VSE). Drug resistance of *S. aureus* strains against cefoxitin and *Enterococcus* strains against vancomycin were investigated both using the minimum inhibitory concentration method (MIC) assay and the SPR system equipped with single and multiple channels. The MIC values of MRSA and MSSA ranged from 32 µg/mL to >128 µg/mL and from 1 µg/mL to 4 µg/mL, respectively. The MIC values of VRE and VSE were between 64 to >128 µg/mL and 2–4 µg/mL, respectively. With the multiple-channel system, the angle shifts of MRSA, MSSA, VRE and VSE were found to be −0.030° and −0.260°, −0.010° and −0.090° respectively. The antibiotic-resistant and susceptible strains were distinguished within 3 h for *S. aureus* strains and within 6 h for *Enterococcus* strains.

## 1. Introduction

Nosocomial infections, which are known as the primary cause of mortality for hospitalized patients, are often acquired by patients during medical care. These infections lead to prolonged hospital stays, a decrease in quality of life, an increase in morbidity and mortality, loss of man-power and increase in health expenditures [1]. According to the World Health Organization, 4,544,100 cases of nosocomial infections influence 4,131,000 patients every year in Europe and cause an economic loss of approximately €7 billion [2]. The rates of nosocomial infections in developed and developing countries are 7% and 10%, respectively [3]. Nosocomial infections, which pose a risk to public health, can be prevented via treatment with antibiotics applied by conscious and correct choice when diagnosed early. Methicillin-resistant *Staphylococcus aureus* (MRSA) and vancomycin-resistant *Enterococcus* (VRE) are the most common hospital pathogens that affect hospitalized and immunocompromised patients.

*S. aureus* gradually develops resistance to the most commonly used antibiotics [4]. As the resistance of *S. aureus* against antibiotics develops, the duration of treatment is prolonged and mortality and morbidity rates increase. Methicillin resistance is the most important because the penicillin-binding protein 2a (PBP2a/PBP2) encoded by *mecA* a genetic marker of resistance to methicillin, shows a low affinity to beta-lactam group antibiotics [5].

Enterococci have a large spectrum of antibiotic resistance [6]. Due to this ability, one of the most common causal agents of nosocomial infections are enterococci, especially *Enterococcus faecium* and *E. faecalis* [7]. *E. faecium* and *E. faecalis*, which carry the resistance genes *vanA* and *vanB*, show vancomycin resistance to glycopeptide antibiotics such as vancomycin and teicoplanin, and these strains are called VRE (vancomycin-resistant *Enterococcus*). VRE constitutes approximately 6% of all *E. faecium* and *E. faecalis* isolates [8].

Antimicrobial agents are generally classified by taking into account the basic mechanism of action consisting of interference with cell wall synthesis (e.g., β-lactams and glycopeptide agents), inhibition of protein synthesis (macrolides and tetracyclines), interference with nucleic acid synthesis (fluoroquinolones and rifampin), inhibition of a metabolic pathway (trimethoprim-sulfamethoxazole), and disruption of bacterial membrane structure (polymyxins and daptomycin) [9]. In our study, cefoxitin and vancomycin, which are members of the β-lactam and glycopeptide antibiotics were used to identify resistant and susceptible strains. The antimicrobial mechanism of cefoxitin occurs by activating bacterial cell autolysins, interfering with the synthesis of a bacterial cell wall and inhibiting the cross-linking of peptidoglycan. As for vancomycin, it hinders the second stage of cell wall synthesis in susceptible bacteria [10].

Various standardized phenotypic antimicrobial susceptibility testing methods such as agar diffusion [11], broth microdilution [12], gradient strip tests [13], and disk diffusion [14] have traditionally been used by researchers to determine whether the bacteria are resistant or susceptible. Resistance to specific antibiotics can also be determined by PCR, which investigates the presence or absence of genes responsible for the resistance [15]. These methods require dedicated microbiology laboratories, specialized personnel, and long analysis time. The disadvantages of antimicrobial susceptibility testing show that new, fast and reliable methods should be developed. The rapid and sensitive detection of pathogenic bacteria using new technologies is a vital issue to improve patient care and to limit nosocomial infections.

Surface plasmon resonance (SPR) biosensors have been used as the dominant method over the years due to their shorter time, reliable and reproducible results, as well as their sensitivity, label-free and real-time detection, and quantitative results [16]. SPR is a phenomenon that measures the formation of surface plasmon polaritons (SPPs) that occur between the interface of a metal and a dielectric substance, typically either a liquid or air. The light beam from a monochromatic, p-polarized light source is reflected from the metal film to a diode-array detector. At that time, oscillations of the conductive electrons called plasmon on the metal surface occur with the wave function of the incident light. The plasmon creates an electromagnetic field, which is referred to as an evanescent wave. As this wave passes from the metal surface to a sample solution, the intensity of the reflected light is called the SPR angle. The SPR angle shift resulting from the molecular interaction is monitored over time [17,18]. Up to the present, SPR application has been used in a variety of research areas including fundamental biological studies, health science research, drug delivery, clinical diagnosis and environmental and agricultural monitoring. The use of SPR-based biosensors has especially increased in pathogen [19,20] and disease detection [21].

Chiang et al. [22] were the first to describe the use of the antimicrobial test for the detection of resistant or susceptible strains using the SPR system. They determined the antimicrobial susceptibility of resistant and sensitive bacteria adhering to a gold chip using a single channel. In another study performed by Syal et al. [23], a single channel was used for an antimicrobial susceptibility test, but a self-assembled monolayer was created to functionalize the sensor surface unlike the previously mentioned study, and the antimicrobial activity of antibiotics was monitored with a transmitted microscope image. Considering studies involving the SPR detection system in the literature, it is observed that the use of multiple channels on the sensor surface is limited to antimicrobial susceptibility testing. Therefore, we focused on constructing multiple microfluidic channels for the simultaneous detection of antimicrobial susceptibility, since the use of multiple channels on the sensor surface has critical importance for monitoring references and multiple samples at the same time. Should the bacteria be susceptible, more SPR angle shifts will occur as the bacteria detach from the gold surface with the antibiotic flow. In other words, the effect of antibiotics on the tested bacteria will be able to be determined by the change in the refractive index. In addition, it will be possible to determine simultaneously which bacteria are resistant or susceptible by monitoring the angle shifts of the negative or positive control in real-time. Therefore, in the present study, the researchers used the SPR technique with single and multiple channel systems to distinguish between methicillin-susceptible and methicillin-resistant strains of *S. aureus* and between vancomycin-susceptible and vancomycin-resistant strains of *Enterococcus*.

## 2. Materials and Methods

### 2.1. Microorganism

Methicillin-resistant *S. aureus* (MRSA) and methicillin-susceptible *S. aureus* (MSSA), vancomycin-resistant *Enterococcus* (VRE) and vancomycin-susceptible *Enterococcus* (VSE), *Staphylococcus aureus* subsp. *aureus* ATCC 43300, *Staphylococcus aureus* subsp. *aureus* ATCC 29213, *Enterococcus faecalis* ATCC 29212, *Enterococcus faecium* B764, *E. coli* ATCC 25922 were obtained from Acibadem Labmed Medical Laboratories (Istanbul, Turkey). The strains of the American Type Culture Collection (ATCC) were used for optimization of the system equipped with a single channel. A total of 40 clinical isolates (MRSA, MSSA, VRE, and VSE) were used to determine their antimicrobial susceptibility in a multi-channel SPR system.

### 2.2. Minimum Inhibitory Concentration (MIC) Assay

All strains were stored with 15% glycerol at −80 °C. Before using the SPR sensing system, broth microdilution method performed in sterile 96-well microplates was applied to all MRSA, MSSA, VRE and VSE strains [24,25,26,27]. Susceptibility to the following 2 antimicrobial agents was tested: cefoxitin for the strains of *S. aureus* and vancomycin for the strains of *Enterococcus* spp. The antibiotics were dissolved in sterile ultra-distilled water and set as a stock solution of 512 µg/mL. The stock solution of antibiotics was serially diluted with Mueller Hinton Broth (Sigma-Aldrich, Darmstadt, Germany) two-fold to 0.5 µg/mL and added to wells apart from control samples. Bacterial suspension without antibiotic was used as a positive control, and the sterile medium was used as a negative control. Each well contained 5 × 10^5^ CFU/mL of the bacterial suspension except for negative controls.

### 2.3. Reagents for SPR System

Poly-L-lysine was used as a gold chip coating material with a concentration of 200 µg/mL. Poly-L-lysine (P8920) solution was obtained from Sigma-Aldrich. Cefoxitin and vancomycin were supplied from Sigma-Aldrich (St. Louis, MO, USA). Both antibiotics were used at a concentration of 3 µg/mL as the minimum level of antimicrobial effect during all antimicrobial susceptibility tests. Sterile ultra-distilled water (DI water) was used as a running buffer throughout all experiments.

### 2.4. Surface Plasmon Resonance Instrument

The surface plasmon resonance system used in our study was manufactured by Nanodev Company (Ankara, Turkey). This portable instrument consists of three components including the sensor, peristaltic pump (Ismatec, ISM597D, Wertheim-Mondfeld, Germany) and the data collection module. A light-emitting diode (LED) was used as an incident light source operating at a wavelength of 850 nm. The high wavelength measurement increased the plasmonic impact area which created a deeper measurement and also increased the sensitivity in bacterial measurements. The cylindrical lens was integrated to collimate light from the LED source by gathering it together and sending it to a rectangular prism (N-BK7, *n* = 1.51). Reflected light from the prism was collected by a CMOS sensor. A circular glass slide with a thickness of 0.15 mm was used to fabricate the chip. A glass slide deposited with 3 nm chrome followed by a 47 nm gold was designed as a disposable microchip. After a refractive index matching oil (*n* = 1.50) was applied to the prism to prevent losses in optical transmission, the gold chip was placed on the center of the prism. An O-ring was attached to the chip to prevent liquid from leaking. The chip was then equipped with a single or multiple microchannel chamber using a screw. A single and multiple microchannel with an inlet and an outlet port were fabricated in order to allow the solution to flow through the surface of the sensor by means of a peristaltic pump. Temperature control was achieved throughout the experiment with a thermistor having a temperature range of 0–60 °C and 0.1 °C temperature control stability. The system was supported by a camera to monitor changes on the chip surface. Recorded images depending on changes of refractive index were converted to resonance angles in real-time by using appropriate custom-designed software.

### 2.5. Preparation of Bacterial Culture for Sensing System

All MRSA, MSSA, VRE and VSE strains were stored at −80 °C with 15% glycerol. The strains were cultured on blood agar (Merck, Darmstadt, Germany, Cat No: 1108860500) supplemented with 5% defibrinated sheep blood (Oxoid, Darmstadt, Germany, Cat No: OXSR0051D) and incubated at 37 °C for 24 h for each experiment. The single colony bacteria were cultured in brain heart broth (BHI, Merck) and incubated overnight at 37 °C in an incubator-shaker (Wisecube^®^WIS-30) for use in the detection system. *E. coli* ATCC 25922 was included as a control.

### 2.6. Modification of Au Surface and Antimicrobial Procedure

**Figure 1:**
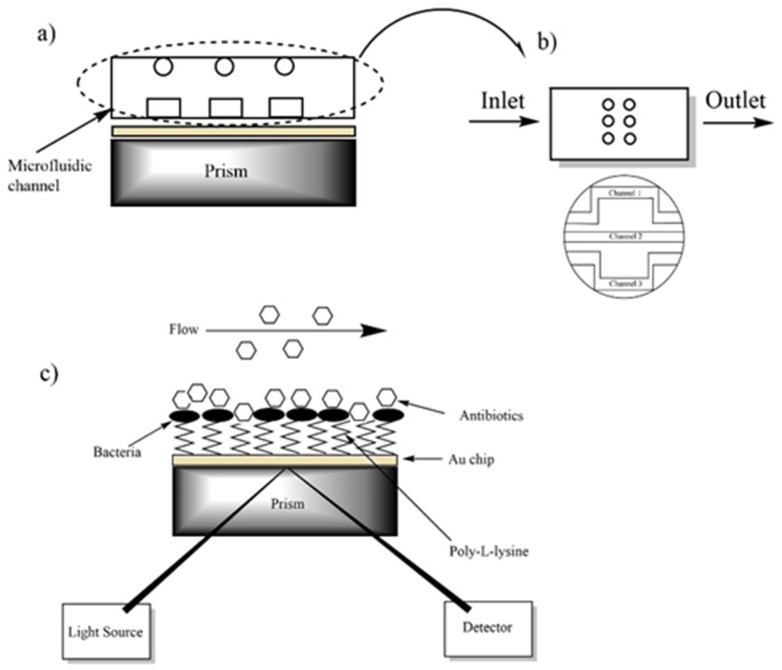
A scheme of the SPR system. (**a**) Illustration of the multiple microfluidic integrated SPR platform. (**b**) The top and bottom appearance of a microfluidic cell chamber. (**c**) Protocol for the antimicrobial test of bacteria.

**Figure 2:**
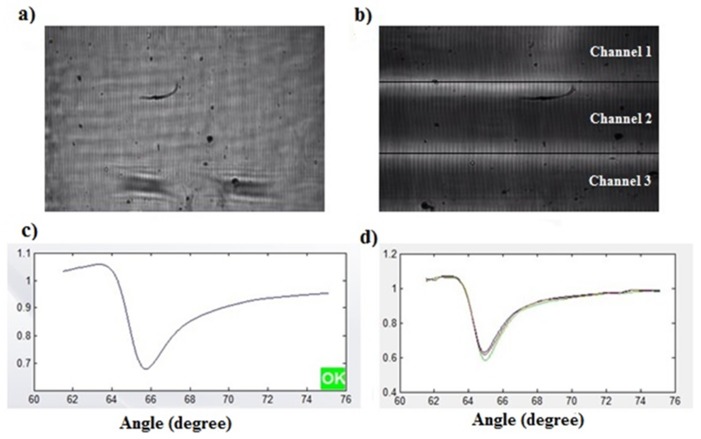
(**a**) The images of the gold surface with the single-cell chamber; (**b**) the images of the gold surface with the triple-cell chamber; (**c**) the resonance angle of DI water on the single- channel; (**d**) the resonance angle of DI water on the triple-channel.

A glass slide coated uniformly with gold to a thickness of 47 nm was used as a sensor chip. Prior to further chemical modification, the gold chip was cleaned in piranha solution which was freshly prepared the ratio of 3:1 (*v*/*v*) mixture of concentrated H_2_SO_4_ and 30% H_2_O_2_ [28] to remove any organic residue from nanofabrication at room temperature for 2 min, and then flushed extensively with DI water and dried in nitrogen flow. The gold chip surface was incubated in 200 µg/mL poly-L-lysine at 4 °C for 24 h [22]. After incubation, the surface was washed with DI water to remove un-coated materials and dried in nitrogen flow. After the surface modification, the gold-coated SPR chip was placed on the glass prism with a thin layer of index-matching oil, and the microfluidic cell chamber was mounted to the SPR instrument with mechanical grippers to ensure continuous flow. The experimental setup for the antimicrobial test of the bacteria is depicted schematically in Figure 1. The modified gold chip surface was imaged by the camera of the system before starting the analysis (Figure 2a,b). To perform the analysis, a steady baseline was created with DI water at a constant flow rate of 25 µl/min, considering the response of the SPR for 30 min as shown in Figure 2c,d. The bacteria for testing, DI water for washing, and the antibiotics for examining of drug resistance were injected to the system using the peristaltic pump, respectively. The binding responses were recorded as an angle degree. SPR angle shifts were calculated by subtracting the values obtained after bacterial binding to those obtained after antibiotic treatment.

### 2.7. Scanning Electron Microscope (SEM) Imaging

SEM imaging was performed using an SEM device (Zeiss, EVO^®^ LS 10) to check the attachment of the bacteria to the surface of the sensor and to compare two methods. The gold chip with bacteria was coated with gold for 60 min to provide better conductivity in SEM. Different magnification levels (from 1.32 KX (KX = 1000X) to 517 X) and voltage of 20,000 kV were used to capture the best images.

## 3. Results

### 3.1. The Results of Minimum Inhibitory Concentration

A total of 40 bacterial strains were evaluated for susceptibility to antibiotics using the microdilution method. The minimum inhibitory concentration (MIC) value of MRSA5 was found as 1 µg/mL. Except for MRSA5, MIC values of the remaining strains of MRSA ranged from 32 µg/mL to >128 µg/mL. As for the MSSA strains, the MIC values of these strains were between 1–4 µg/mL excluding for MSSA1 and MSSA10. The MIC of VRE strains ranged from 64 to >128 µg/mL, while the MIC of VSE strains was between 2–4 µg/mL. After determination of the MIC ranges of the strains, these clinical isolates were used for further analysis of the SPR system.

### 3.2. Determination of Antimicrobial Susceptibility of MRSA and MSSA with a Single Microfluidic Channel

**Figure 3:**
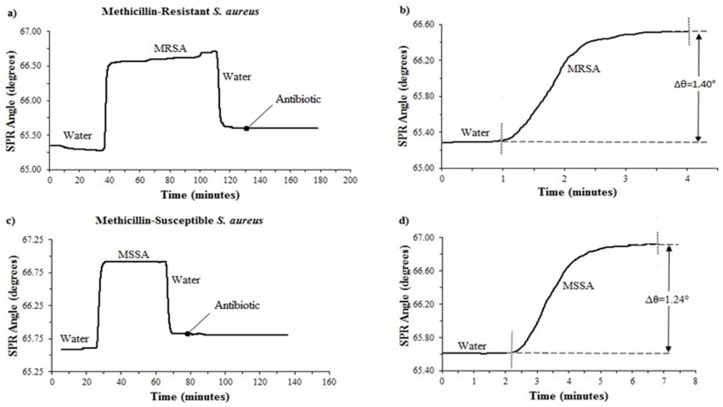
SPR angle shift of methicillin-resistant (the strain of MRSA 2) and susceptible *S. aureus* (the strain of MSSA 7): (**a**) Kinetic plot of SPR angle shift of methicillin-resistant *S. aureus* to cefoxitin antibiotic for 60 min; (**b**) SPR response of resistant bacterial insertion; (**c**) kinetic plot of SPR angle shift of methicillin-susceptible *S. aureus* to cefoxitin antibiotic for 60 min; (**d**) SPR response of susceptible bacterial insertion.

To optimize the system equipped with a single-channel, *Staphylococcus aureus* subsp. *aureus* ATCC 43300 and *Staphylococcus aureus* subsp. *aureus* ATCC 29213 were used. A total of 10 MRSA and 10 MSSA were then tested with the SPR system. The SPR angle shifts of antibiotic-resistant and susceptible *S. aureus* are shown in Figure 3. A baseline was firstly created by injecting DI water. The association phase started with an injection of bacteria to allow binding. The peristaltic pump injected the bacteria onto the sensor surface. When MRSA and MSSA were attached to the surface, 1.40° ± 0.523853° and 1.24° ± 0.570678° angle shifts occurred, respectively (Figure 3b,d). Once the association phase was completed, DI water was primed to the system to remove loosely bound materials for the dissociation phase. The rapid decrease of the SPR angle in the washing process was clearly seen in Figure 3a,c. The SPR angle did not reach DI water baseline level, indicating that the bacteria were successfully bound. After achieving a constant binding response at the surface, the antibiotic solution was injected into the system. The SPR angle shifts were recorded against time during all experiments.

Our experimental results showed that after 60 min cefoxitin (3 µg/mL in DI water) treatment, the SPR angle shifts were −0.0280° ± 0.000735° for susceptible strains and −0.00072° ± 0.001383° for resistant strains.

### 3.3. Determination of Antimicrobial Susceptibility of VRE and VSE with a Single Microfluidic Channel

**Figure 4:**
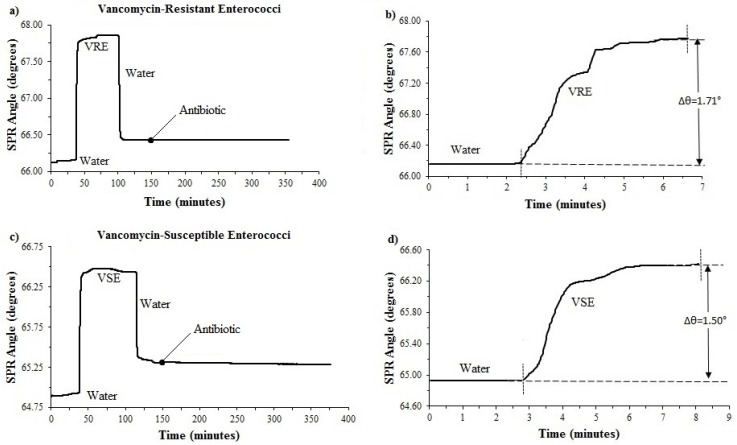
SPR angle shift of vancomycin-resistant (strain VRE 5) and susceptible enterococci (strain VSE 8): (**a**) Kinetic plot of SPR angle shift of vancomycin-resistant enterococci to vancomycin antibiotic for 200 min; (**b**) SPR response of resistant bacterial insertion; (**c**) kinetic plot of SPR angle shift of vancomycin-susceptible enterococci to vancomycin antibiotic for 200 min; (**d**) SPR response of susceptible bacterial insertion.

*Enterococcus faecalis* ATCC 29212 and *Enterococcus faecium* B764A were used to determine the optimum conditions for the system equipped with a single-channel. A total of 10 VRE and 10 VSE were then tested on the single-channel system. For the surface-binding of bacteria experiment, DI water was first injected to the SPR system via a peristaltic pump and then measurements were made to test the adhesion of the VRE and VSE isolates to the poly-L-lysine (200 μg/mL) coated surface. The introduction of bacteria to the system resulted in a signal increase, 1.71° ± 0.697409° for the resistant strain, 1.50° ± 0.662909° for the susceptible strain (Figure 4b,d). After binding of the bacteria to the poly-L-lysine on the chip, the washing with DI water was performed to remove unbound bacteria. It was discovered that the SPR angle shifts were −0.02489° ± 0.008638° for susceptible strains and −0.00033° ± 0.000261° for resistant strains after 200 min vancomycin (3 µg/mL in DI water) treatment (Figure 4a,c). A ten-fold difference was found between susceptible and resistant *Enterococcus* spp.

### 3.4. Determination of Antimicrobial Susceptibility of MRSA and MSSA with a Triple Microfluidic Channel

**Figure 5:**
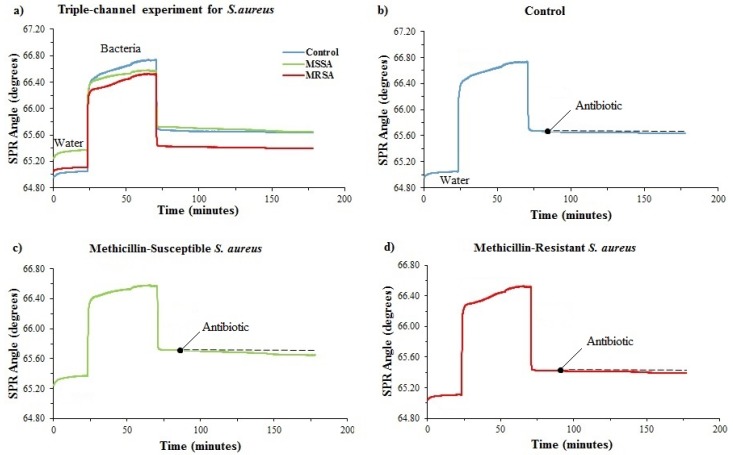
(**a**) Real-time SPR kinetic curve for antibiotic-resistant and susceptible *S. aureus* against cefoxitin treatment on 3-channel system. (**b**) Control sample, *E. coli*. (**c**) Methicillin-susceptible *S. aureus* (the strain of MRSA 2). (**d**) Methicillin-resistant *S. aureus* (the strain of MRSA 2).

The triple microfluidic channel was used for simultaneously observing SPR angle shifts of antibiotic-resistant and susceptible *S. aureus*. The results obtained are shown in Figure 5a. The data obtained as angle degree from the system was converted into individual graphics to make the graph more understandable (Figure 5b–d). As we did on a single channel, the same process steps were performed on a triple microfluidic channel. After creating a baseline with DI water, cefoxitin was introduced to the surface for ~100 min. The SPR angle shifts were −0.260° ± 0.019326° for susceptible strains and −0.030° ± 0.008769° for resistant strains and −0.090° ± 0.007347° for *E. coli* used as a control sample.

### 3.5. Determination of Antimicrobial Susceptibility of VRE and VSE with a Triple Microfluidic Channel

**Figure 6:**
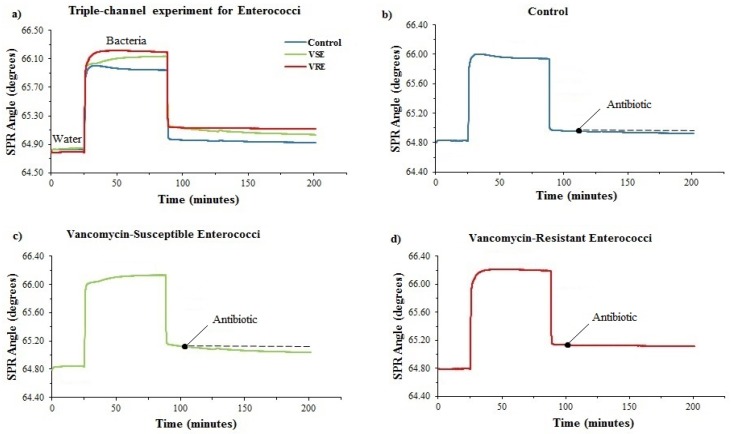
(**a**) Real-time SPR kinetic curve for antibiotic-resistant and susceptible enterococci against vancomycin treatment on the 3-channel system. (**b**) Control sample, *E. coli*. (**c**) Vancomycin-susceptible enterococci (strain VSE 8). (**d**) Vancomycin-resistant enterococci (strain VRE 2).

To perform antimicrobial susceptibility test of *Enterococcus* against vancomycin in a triple microfluidic chamber, the poly-L-lysine-coated chip was mounted into the system prior to the analysis. The same procedure described above was then applied to all *Enterococcus* strains. Vancomycin was delivered to the gold chip surface for 100 min at 25 μL/min. The measurement results of *Enterococcus* conducted with the triple microfluidic channel are shown in Figure 6. It was discovered that the total reflectivity changes of *Enterococcus* were −0.090° ± 0.006611° for susceptible strains, −0.010° ± 0.003734° for resistant strains and −0.050° ± 0.011958° for *E. coli* used as a control sample.

### 3.6. SEM Imaging

**Figure 7:**
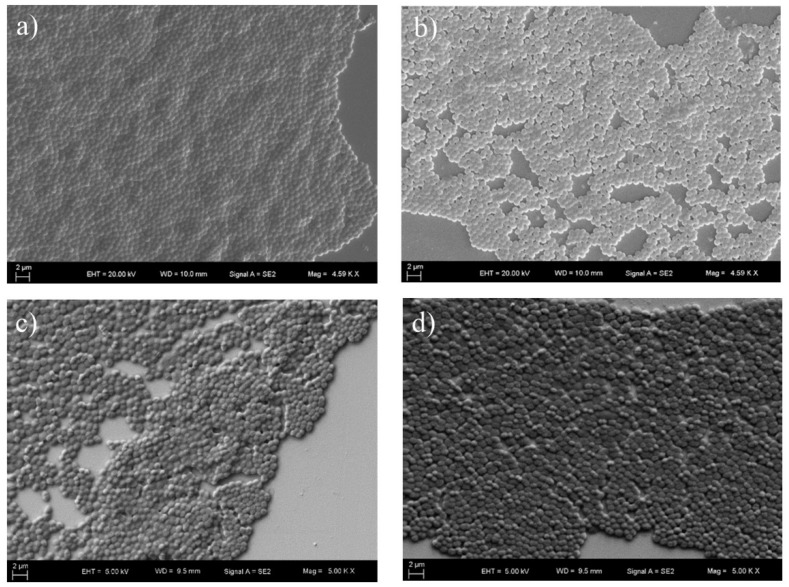
SEM micrographs of the bacteria without antibiotic treatment. (**a**) Methicillin-resistant *S. aureus* at 4.59 KX magnification; (**b**) methicillin-susceptible *S. aureus* at 4.59 KX magnification; (**c**) vancomycin-resistant enterococci at 5 KX magnification; (**d**) vancomycin-susceptible enterococci at 5 KX magnification.

**Figure 8:**
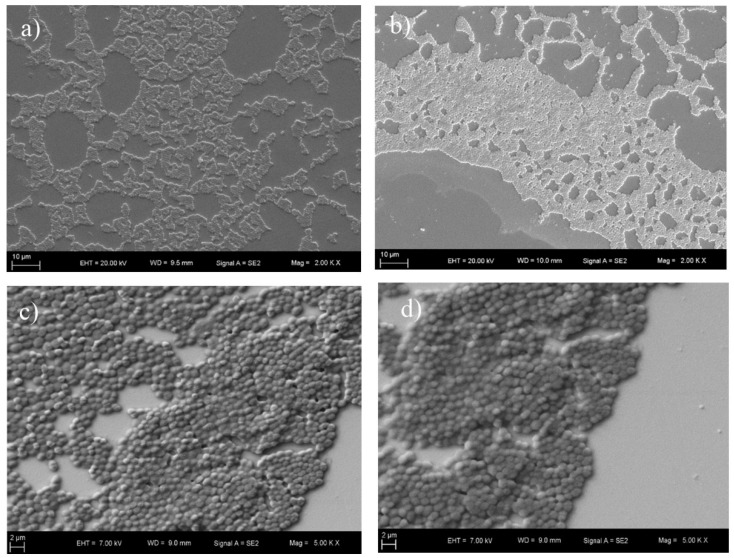
SEM micrographs of the bacteria with antibiotic treatment. (**a**) Methicillin-resistant *S. aureus* at 2 KX magnification; (**b**) methicillin-susceptible *S. aureus* at 2 KX magnification; (**c**) vancomycin-resistant enterococci at 5 KX magnification; (**d**) vancomycin-susceptible enterococci at 5 KX magnification.

SEM micromorphology of sensor chips with and without antibiotic treatment was analyzed to observe the presence and the attachment of MRSA, MSSA, VRE and VSE. The SEM images are shown in Figure 7 and Figure 8 for the MRSA, MSSA, VRE and VSE strains. There was no significant difference between the images obtained after antibiotic treatment and those obtained without antibiotic treatment. This result showed that the SPR system received more accurate measurements than the SEM device. SEM results also demonstrated that bacteria were successfully bound to the poly-L-lysine-coated surface.

## 4. Discussion

In the first part of the study, we examined all strains using the MIC method to determine the susceptibility to antibiotics. According to the Clinical and Laboratory Standards Institute [29], the breakpoint of *S. aureus* to cefoxitin is ≤4 µg/mL for susceptible strains and ≥8 µg/mL for resistant strains, and the breakpoint of *Enterococcus* spp. is ≤4 µg/mL for susceptible strains and ≥32 µg/mL for resistant strains, respectively. In this study, the MIC values of MRSA were 32 µg/mL to ≥128 µg/mL except for MRSA5. On the other hand, the MIC values of MSSA were between 1–4 µg/mL except for MSSA1 and MSSA10. The high MIC values can be interpreted as an indication that these two strains have the ability to produce excessive beta-lactamase, which causes resistance. The results of a study conducted by von Ah et al. [30] showed that MIC values of MSSA and MRSA were 4 mg/L and 32 mg/L, respectively. The results of our study are consistent with their results. In the present study, the findings for the MIC values of VRE and VSE are also in concordance with data reported by Schouten et al. [31] and CLSI [29].

Herein, we developed an online simultaneous method to test the condition of antibiotic-resistant bacteria instead of using traditional methods. Briefly, the innovative aspect of this work is a locally manufactured SPR system and a multi-channel design for this system. The surface of the gold chip is divided into three sections with a multiple microfluidic channel. The surface of a chip surface was simultaneously immobilized in real-time. To be successful on a multiple microfluidic channel, experiments were first tested on a single channel. Angle shifts of all experiments showed that the difference between resistant and susceptible strains was about ten-fold or more. Similar results were also documented in a study by Chiang et al. [22] who reported a ten-fold difference between ampicillin-susceptible and resistant strains of *E. coli* JM109.

Antibiotic treatment was found to cause changes in the structure of the bacterial cell wall of susceptible strains and decrease the refractive index. The resistant strains had an almost constant angle during antibiotic treatment compared to susceptible strains. This demonstrated that the angle shifts of susceptible strains were due to inhibition of cell wall synthesis by antibiotics. On the other hand, the change of SPR signal obtained after addition of antibiotic looks subtle, since the bacteria have a great SPR angle shift. Therefore, whether the bacteria is affected by the antibiotic flow can be determined by the results obtained after the calculation.

The testing time of the present study was less than 24 h to detect MRSA, MSSA, VRE and VSE. Scholars have used different methods to investigate MRSA and VRE strains [32,33]. The most common method among these methods has been the broth microdilution method [34]. However, this method requires a longer testing time than our study. Cansizoglu et al. [35] introduced a novel platform for rapid ultra-sensitive detection of antimicrobial susceptibility. In that study, the minimum inhibitory concentration of bacteria was measured within 2 to 4 h by using a rapid ultra-sensitive detector (RUSD) utilizing changes in refractive index between two media with a different refractive angle. In another study, a BD (Becton Dickinson) phoenix system based on rapid identification and antimicrobial susceptibility testing of gram-positive cocci in blood cultures was used by Lupetti et al. [36]. The testing time of that study was 12–24 h earlier than the current method conducted with that device. Our results are consistent with the findings of these two studies.

Integrated multi-channel platforms offer the potential to reduce the cost of diagnostic platforms [37]. Since our multi-channel platform divides a chip surface into three or more sections, multi-detection can be conducted on a single chip. With this analytical approach, this platform can be used not only in antimicrobial susceptibility testing but also in other clinical detection methods by using different immobilization methods. Furthermore, a single microorganism can undergo multiple antibiotic tests simultaneously with our 3-channel system.

Compared with the traditional methods, our results show that the SPR platform is faster, and requires less labor, than other methods used in the literature [38]. On the other hand, abnormalities in bacteria adherence to the substrate and adhesion stability are the disadvantages of the SPR system. In this study, bacterial culture was incubated in an incubator, and then used in the system to monitor the behavior against the antibiotic. In the future study, those two steps might be combined and examined in real-time. To do this, multiple holes might be designed instead of a flowing system. In addition, decreasing bacterial counts can be calculated by quantitative analysis.

## 5. Conclusions

Nosocomial infections, also known as hospital-acquired infections (HAIs), represent a significant risk to public health, as they are responsible for significant morbidity and mortality. If the early diagnosis of infections is performed, the antibiotic treatment applied with conscious and correct selection will be successful in the treatment of hospitalized patients. For these reasons, the rapid identification of bacteria causing hospital infections is important for appropriate treatment. In this study, we demonstrated the use of an antimicrobial susceptibility test to rapidly detect resistant or susceptible strains. MRSA and VRE strains had an almost constant SPR angle during antibiotic flow. Since cefoxitin and vancomycin hinder bacterial cell wall synthesis, MSSA and VSE strains exhibited a regular SPR angle decrease. Resistant and susceptible strains could be detected using SPR in less than 12 h (3 h for MRSA and MSSA, 6 h for VRE and VSE) and could be used directly in the system since they did not require dilution for testing.

In conclusion, the most important aspect of our study is that the antimicrobial susceptibility test was carried out with a developed multi-channel system. With this system, multiple analyses were performed simultaneously with a control sample. Our study indicates that the current system provides an alternative to conventional detection techniques for antimicrobial susceptibility testing.
